# RNA binding proteins are potential novel biomarkers of egg quality in yellow catfish

**DOI:** 10.1186/s12864-023-09220-9

**Published:** 2023-03-16

**Authors:** Fan Ren, Quan Zhou, Yinglu Meng, Wenjie Guo, Qin Tang, Jie Mei

**Affiliations:** 1 College of Fisheries, Huazhong Agricultural University, Wuhan, 430070 China; 2 Hubei Hongshan Laboratory, Wuhan, 430070 China

**Keywords:** Egg quality, RNA-binding proteins, Gene expression, Biomarker

## Abstract

**Background:**

Egg quality is a major concern in fish reproduction and development. An effective evaluation of egg quality prior to fertilization is helpful in improving the fertilization rate and survival rate of the larva. In this study, we aim to identify quality instructors from the combination study of fertilization rate, hatching rate, embryo malformation rate and gene expression profile.

**Results:**

Eggs from 25 female fish were fertilized with sperm from the same fish. The egg quality was determined by the fertilization rates, hatching rate and embryo malformation rate and divided into three categories, low-quality (< 35%), medium-quality (35 to 75%), and high-quality (> 75%). Due to the distinct difference in fertilization, hatching and embryo malformation rate between low-quality eggs and high-quality eggs, these two groups were considered for the identification of quality markers. Then RNA-seq was performed for the originally preserved eggs from the low-quality group and high-quality group. We profiled the differentially expressed genes and identified a group of RNA-binding proteins (RBPs) as potential regulators. Gene function analysis indicated that most of these genes were enriched in RNA-regulated pathways including RNA processing. The RBPs were more related to egg quality from the PLS-DA analysis. Finally, gene expression was validated by qRT-PCR.

**Conclusions:**

We found a cluster of RBP genes including *igf2bp3, zar1*, *elavl1*, *rbm25b* and related regulatory factors including *yy1*, *sirt1*, *anp32e*, *btg4* as novel biomarkers of egg quality.

**Supplementary Information:**

The online version contains supplementary material available at 10.1186/s12864-023-09220-9.

## Background

The reproductive performance of fish is determined by its genetic characteristics (gene expression, RNA abundance, etc.) and environmental factors (temperature, light, nutrients, etc.), and regulated by internal factors such as hormones and endocrine factors through the hypothalamic-pituitary-gonadal axis [1]. Egg quality is also an important factor affecting fish reproduction by directly related to the fertilization rate, development of embryos, and survival rate of juveniles [2]. Many studies have confirmed that endogenous and exogenous factors can affect egg quality [3–5]. However, the molecular and cellular mechanisms regulating egg quality are still unclear, and the evaluation criteria for egg quality have not been defined in catfish. Therefore, to promote healthy breeding, the essential work is to summarize the influencing factors and screen out egg quality markers.

There are diversities of evaluation criteria for egg quality. Preliminarily, it can be evaluated from the parameters of egg morphology, biochemical composition, fertilization rate, hatching rate and embryo malformation rate [1]. Many studies have confirmed that for some teleosts, the quality of eggs can be reflected by morphology such as diameter, surface color, transparency, buoyance, shape and distribution of oil globules [6–10]. For example, egg diameter can be used as an essential indicator to determine egg activity for turbot (*Scophthalmus Maximus*) [11]. When the egg diameter is 0.9 ~ 1.1 mm, it can produce a high fertilization rate, a larger egg diameter does not represent a higher fertilization rate and hatchability [11, 12]. Biochemical composition is also an indicator of egg quality. Lahnsteiner et al. (2005) showed that the shape and size of lipid droplets in eggs could affect the survival rate of young bream [13]. Mansour et al. (2007) studied brown trout and found that the distribution of lipid droplets in eggs could affect the development of embryos [8]. However, Ciereszko et al. (2009) applied the same method to evaluate the quality of eggs from cultured rainbow trout, but it had no significant effect [14]. Therefore, some researchers believe that embryonic development after fertilization can more accurately reflect the quality of the egg [1, 15]. The fertilization rate indicated by the proportion of abnormal embryos and malformed larvae is another evaluation criterion of egg quality. After fertilization, embryonic cells undergo cleavage, previous study on Atlantic cod (*Gadus morhua*) indicated that low-quality eggs resulted in abnormal cleavage of zygote and caused the death of early embryos [16]. Besides, the development status of embryos at various stages is often used to evaluate the quality of eggs, such as the gastrula stage, incubation stage and yolk absorption stage [2].

With the continuous development of molecular biology, the detection of egg quality at the molecular level has been widely carried out. For example, cytokines and growth factors play synergistic roles in the regulation of oogenesis and early embryonic development of mammalian [17, 18]. Except for endocrine factors, investigation of maternal RNA levels is also a consideration to find indicators for evaluating oocyte quality in the teleost [19, 20]. To our knowledge, the stability of maternal RNAs deposited in oocytes can indicate the healthy development of embryos. A defective turnover of maternal RNA results in the failure of oocyte maturation and embryogenesis. Among these maternal factors, RNA-binding proteins can be considered as critical determinants of egg quality in mice and zebrafish [21, 22]. The disruption of RNA-binding protein stability led to severe developmental defects in zebrafish and medaka [23–26]. Besides, some maternal mRNA regulators were also revealed as biomarkers of oocyte development by facilitating the maternal mRNA degradation, loss of them cause female infertility and impaired degradation of maternal mRNA in oocytes [27–30]. According to these studies, it is valid to infer that RNA-binding proteins potentially regulate egg quality by controlling maternal RNA abundance. It provides us insights into identifying markers from RNA-binding proteins through a comparison of high-quality eggs and low-quality eggs prior to fertilization.

From the above, a combined assessment of morphology, fertilization rates, hatching rate and embryo malformation rate and expression abundance of biomarkers is a comprehensive consideration to improve the quality of eggs and optimize breeding. In this study, we evaluated the egg quality of yellow catfish by investigating the fertilization, hatching and embryo malformation rate and profiled the transcriptome of high-quality and low-quality eggs. By comparing the abundance of RNA-binding proteins, we identified biomarkers related to egg quality. In general, identification of quality markers from eggs and evaluation of their abundance will help us promote fertilization and create a sustainable development of fish culture.

## Results

### Assessment of egg quality and RNA-seq for high and low-quality eggs

**Fig. 1:**
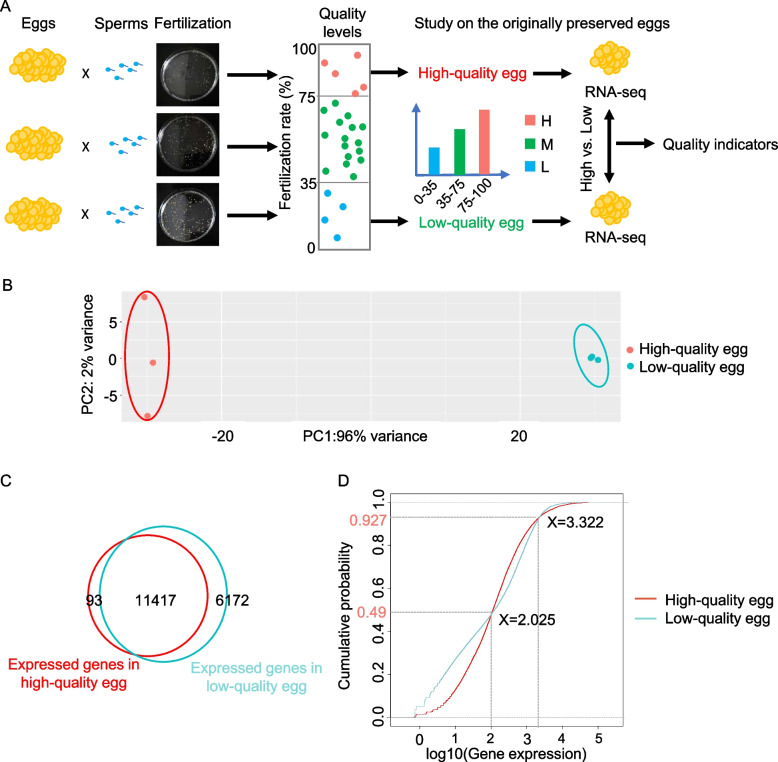
Schematic pipeline of fertilization, evaluation of egg quality and RNA-seq for originally preserved eggs of yellow catfish. **A** Sampling of eggs and sperm, fertilization, statistic of fertilization rate, evaluation of egg quality, and RNA-seq for originally preserved eggs. **B** Principal component analysis (PCA) of RNA-seq samples. **C** Overlapping of genes expressed in high-quality egg and low-quality egg. **D** The cumulative distribution of genes in high-quality egg and low-quality egg. The x-axis indicates expression level of genes and y-axis indicates the cumulative percentage of genes. From left to right, gene expression level increases gradually, while from bottom to up the cumulative percentage of genes increases gradually

To estimate the developmental potential of eggs, evaluating egg quality is critical before fertilization. In addition to the estimation by size and morphology, the success rate of fertilization, hatching rate and malformation rate are also criterion for evaluating the quality. In this study, we sampled eggs from 25 female yellow catfish and fertilized them with sperm from the same male fish, respectively. For each group, the fertilization rate, hatching rate and malformation rate were calculated. The quality of eggs was classified into three groups according to these indicators. In detail, the low-quality group with a fertilization rate lower than 35%, the medium-quality group with a fertilization rate from 35 to 75%, and the high-quality group with a fertilization rate higher than 75%, respectively (Fig. 1A). The hatching rate increased while the malformation rate decreased from the low-quality group to high-quality group (Fig. S1). To dissect quality indicators, the low and high-quality groups were selected for further investigation due to the big difference between these rates. The originally preserved eggs from three female yellow catfish of each group were selected for RNA-seq. Principal component analysis (PCA) of samples showed that three biological replicates clustered together and the low and high-quality groups were distinguished from each other (Fig. 1B). A total of 17,589 genes were detected from the low-quality group while 11,510 genes were detected from the high-quality group, of which 11,417 genes were commonly expressed through overlapping the two gene sets, along with 93 high-quality-egg-specific and 6172 low-quality-egg-specific expressed genes (Fig. 1C). To investigate the relationship between the change in gene expression and the increasing ratio of gene numbers, the cumulative distributions of genes in the two groups were detected (Fig. 1D). We used red and turquoise curves to represent gene distributions of different groups. In the figure, 49% of genes indicated expression levels lower than 2.025 (log_10_FPKM). For this 49% of genes, the line of low-quality eggs lies above the line of high-quality eggs, which means an overall lower expression level in the low-quality group. While about 43.7% of genes indicated expression levels between 2.025 and 3.322. Generally, at these genes, the low-quality group showed a higher expression level than the high-quality group. Besides, 7.3% of genes are highly expressed with log_10_FPKM greater than 3.322. Above this expression level, the low-quality group showed a lower expression level than the high-quality group. In general, the overall gene expression of low-quality eggs was slightly lower than high-quality eggs.

### Gene expression differences between low and high-quality eggs of yellow catfish

**Fig. 2:**
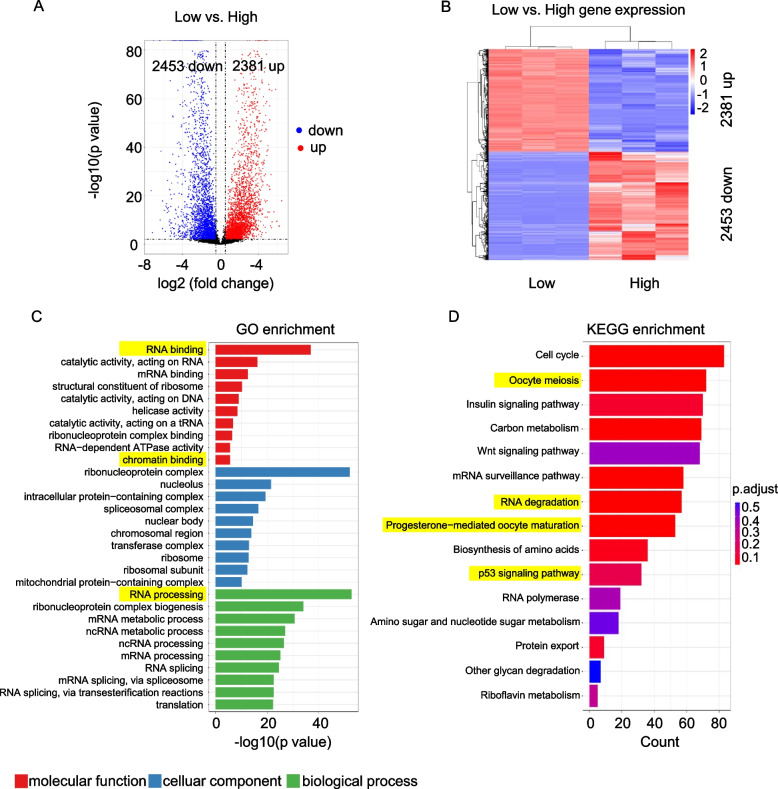
Identification of differentially expressed genes from low-quality eggs vs. high-quality eggs. **A** Volcano plot of differential genes identified from low-quality eggs vs. high-quality eggs. **B** Heatmap showing DEG expression pattern. **C** Functional annotation of DEGs, enriched GO terms. **D** Functional annotation of DEGs, enriched KEGG pathways

Profiling gene expression difference between low and high-quality eggs is an effective way to detect quality indicators. In this study, we identified 4834 differentially expressed genes (DEGs) from low-quality vs. high-quality eggs, including 2453 down and 2381 upregulated genes (Fig. 2A). Although the number of downregulated genes was close to that of upregulated genes, a slight difference could still be caught, which was consistent with the conclusion from the overall cumulative distribution that the low-quality genes are more than high-quality genes. The expression pattern of DEGs is shown in Fig. 2B. Function annotation of the total DEGs was performed by GO analysis and KEGG pathway enrichment. The top-ranked terms from the biological process, molecular function, and cellular components were listed in Fig. 2C, and the KEGG pathways were listed in Fig. 2D. Among these functions, DEGs enriched RNA and oocytes-related activities were marked in yellow, which indicates that the mRNA levels of eggs are potentially correlated with egg quality. Our results indicated that the process of RNA degradation and oocyte meiosis were essential for the egg quality maintenance.

### Annotation and validation of downregulated genes

**Fig. 3:**
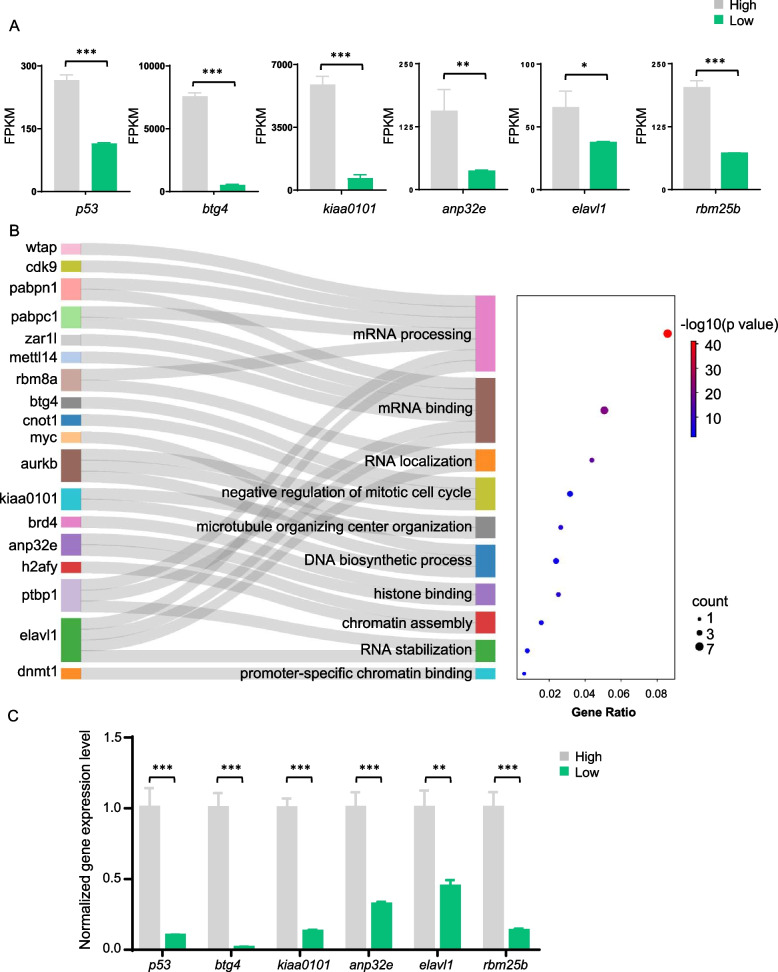
Validation of downregulated genes. **A** Expression levels (FPKM) of *p53*, *btg4*, *p15*, *anp32e*, *elavl1*, *rbm25b* in low and high-quality eggs. **B** Sankey diagram showing significantly downregulated genes in low-quality eggs and related processes they were involved in. **C** Validation of *p53*, *btg4*, *p15*, *anp32e*, *elavl1*, *rbm25b* gene expression by qRT-PCR

To find genes that indicate egg quality, we further annotated the downregulated genes and upregulated genes, respectively. The genes downregulated in low-quality eggs and upregulated in high-quality eggs were significantly enriched at the GO terms of mRNA binding, mRNA 3′-UTR binding, mRNA processing, and chromatin-related process (Fig. S2A). Meanwhile, the KEGG results displayed that these genes also enriched in ovarian development and egg quality-related pathways including oocyte meiosis, oocyte maturation and the p53 signaling pathway (Fig. S2B). These pathways were all involved in ovarian development and egg quality. Next, we checked the expression of genes including *p53*, *btg4*, *p15*, *anp32e*, *elavl1* and *rbm25b* from RNA-seq (Fig. 3A). As a result, these genes played fundamental functions in maternal RNA degradation, RNA stability and splicing and chromatin regulation to regulate oocyte and embryo development, were all downregulated in the low-quality eggs. Except for these genes, the Sankey diagram showed more downregulated genes enriched at the mRNA processing, mRNA binding, chromatin assembly and some potential egg quality control pathways, such as *wtap*, *pabpn1*, *rbm8a*, *kiaa0101* (Fig. 3B). Gene expression were validated by qRT-PCR (Fig. 3C), which were in keeping with the RNA-seq gene expression.

### Annotation and validation of upregulated genes

**Fig. 4:**
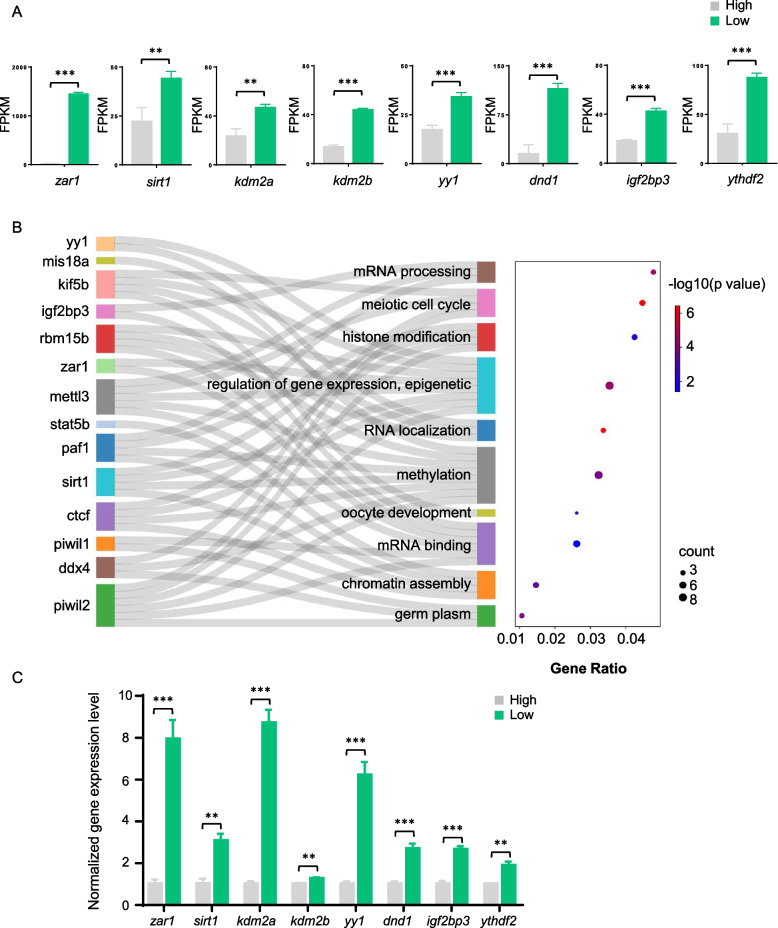
Validation of upregulated genes. **A** Expression levels (FPKM) of *zar1*, *sirt1*, *kdm2a*, *kdm2b*, *yy1*, *dnd1*, *igf2bp3*, *ythdf2* in low and high-quality eggs. **B** Sankey diagram showing the upregulated genes in low-quality eggs and the corresponding pathways they were involved in. **C** Validation of *zar1*, *sirt1*, *kdm2a*, *kdm2b*, *yy1*, *dnd1*, *igf2bp3*, *ythdf2* gene expression by qRT-PCR

We also investigated the significantly upregulated genes in low-quality eggs. Gene annotation indicated that most of these genes were enriched in RNA-related processes, such as RNA binding and RNA degradation via the CCR4-NOT4 complex (Fig. S2C). KEGG terms showed that these genes were enriched in the cell cycle, oocyte meiosis, and RNA-degradation pathways, which are essential for maintaining normal oogenesis (Fig. S2D). These downregulated functions undoubtedly indicated the low quality of eggs. Some of the upregulated genes from these pathways were subsequently investigated, including *zar1*, *sirt1*, *kdm2aa*, *kdm2ab*, *yy1*, *dnd*, *igf2bp3*, *ythdf2* (Fig. 4A). These factors including RNA binding proteins and epigenetic regulation factors mediated oocyte development and oocyte meiotic maturation by regulating maternal mRNA degradation and transcription. Interestingly, the expression levels of the RNA-binding proteins Igf2bp3 and Ythdf2, m^6^A readers regulating maternal RNA turnover in the processes of oocyte and embryo development, were also significantly reduced in the high-quality eggs. Moreover, our result showed that only Igf2bp3 but not Igf2bp1 and Igf2bp2 were differentially expressed between the low and high-quality eggs. This indicated that Igf2bp3 might play a special role in egg quality by maintaining maternal RNA stability. In this study, our findings indicate that Igf2bp3 may be a potential marker for oocyte development in yellow catfish. Its upregulation could result in defective enrichment of mRNA in oocytes or the lower quality of eggs. Except for the genes investigated above, the Sankey diagram also showed that some other genes such as *rbm15b*, *stat5b* and *ddx4* affect the functions mentioned above (Fig. 4B). And the gene expression was validated by qRT-PCR (Fig. 4C). In general, eggs displayed low quality due to the dysregulation of RNA-binding proteins and abnormal enrichment of RNAs in eggs, RNA stability and degradation were destroyed in the maturation of eggs.

### RNA-binding protein (RBP) functions in the maturation of eggs and its role in controlling egg quality

**Fig. 5:**
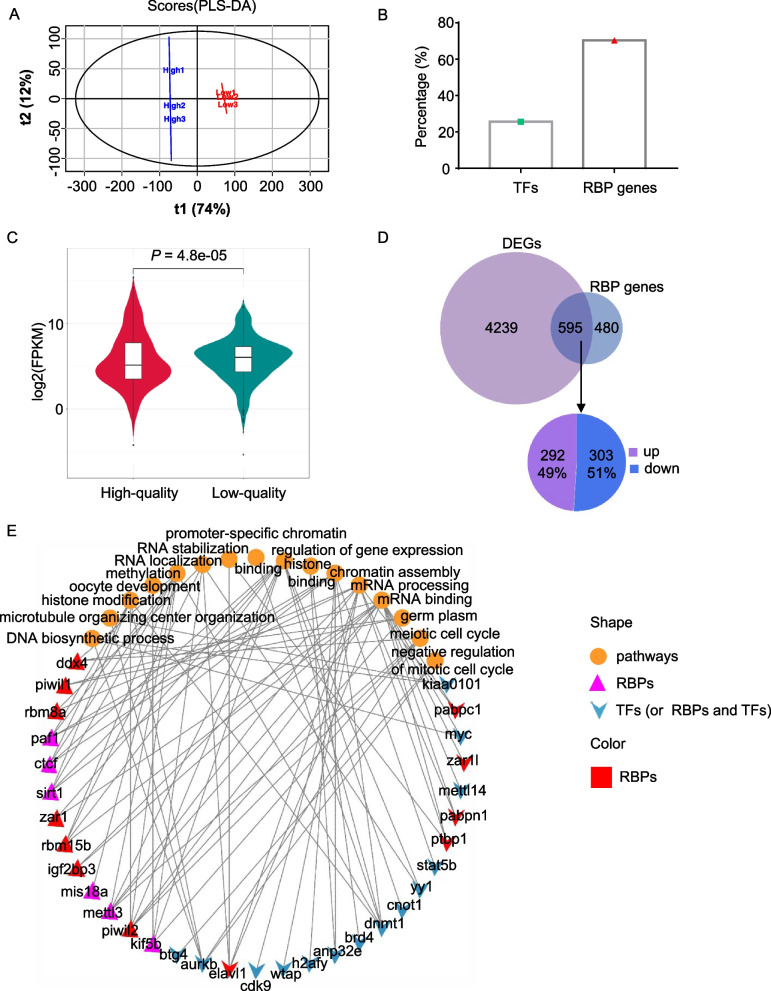
RNA-binding protein (RBPs) functions in the maturation of eggs and its role in controlling egg quality. **A** Screening of genes that contributed to the distinction of low and high-quality eggs. Genes were ranked by VIP-score generated from PLS-DA analysis, the threshold was set as VIP-score > 1.0, a total of 5517 genes were obtained and used to draw the fig. **B** Among the screened genes, 70.3% are RBP genes while 25.6% are transcription factors (TFs). **C** Violin plot of RBP gene expression in high and low-quality eggs. **D** Overlapping of RBP genes and DEGs, statistics of up and downregulated genes. **E** RBP genes and other regulatory factors (such as TFs) enriched the pathways

From the above analysis, RNA-binding proteins play essential roles in controlling RNA abundance and regulating egg quality. Here, we investigated all the RBP genes of the yellow catfish genome. Firstly, through OPLS-DA (Orthogonal Partial Least-Squares Discriminant Analysis), totally we obtained 5517 genes with VIP-score > 1.0. The VIP score is a measurement of the importance of genes in the OPLS-DA model. A higher VIP score usually indicates that the gene is more important. As a result, the low-quality group and high-quality group were clearly distinguished by the 5517 genes (Fig. 5A). We further calculated the proportion of RBP genes in the 5517 genes and found that about 70.3% of the genes are RBP genes, about 25.6% are transcription factors (TFs), and the rest are common genes without regulatory functions (Fig. 5B). We also compared the expression levels of RBP genes between low-quality and high-quality eggs and found that it was slightly higher in the low-quality group (Fig. 5C). This result means that in low-quality eggs the higher expression of RBPs destroyed the stability of RNA abundance.

Next, we focused on a group of RBP genes differentially expressed between low-quality and high-quality eggs. Through overlapping the global DEGs with RBP genes, we got 595 differential-RBP genes, of which 49% were upregulated and 51% were downregulated (Fig. 5D). The functions of these up and downregulated genes are shown in Fig. S3. And crosstalk analysis indicated that some differentially expressed RBP genes including *igf2bp3*, *zar1*, *elavl1*, *rbm25b* and other regulatory factors including *anp32e*, *btg4, sirt1*, *yy1* are working together to affect pathways such as RNA binding, mRNA processing, regulation of gene expression, RNA stabilization, and chromatin assembly (Fig. 5E). These network genes could be taken as potential indicators to distinguish the low and high-quality eggs.

## Discussion

RNA level is dynamically determined by the RNA activation and degradation during oocyte development. The stabilization of RNA abundance is essential for the developmental events of oogenesis and subsequent embryogenesis. Although previous studies have focused on the transcriptomic gene expression during the oogenesis of catfish [20, 31], whether RNA abundance and gene expression stability can be used as a criterion of egg quality is still unclear. In this study, we evaluated egg quality levels by fertilization rate, hatching rate and embryo malformation rate to detecte the transcriptomic differences between high-quality and low-quality eggs. We found that the RNA-binding proteins were related to RNA abundance, gene expression stability and oogenesis, some of them could be considered as potential biomarkers of egg quality.

In this study, although all fish were selected from the same genetic background and kept under the same condition, the quality of eggs varied among individuals. Previous study reported that the reproductive performance of fish is not only determined by its genetic characteristics and environmental conditions but also regulated by some internal factors such as hormones and endocrine factors [32–34]. Besides, the feed composition, digestion, conversion rate and growth rate also affected the reproductive system development of fish, such as primordial germ cell (PGC) formation, oogenesis and egg maturation [2, 4]. Nevertheless, the variation of individuals in this study allows us to study differences in egg quality and look for markers indicating egg quality. By dividing the fertilization rate, hatching rate and embryo malformation rate into three levels, we obtained the high and low-quality eggs and performed RNA-seq analysis. Interestingly, we got more genes expressed in low-quality eggs, but the expression level is lower than the high-quality group globally, which indicated an instability of RNA activation and degradation in low-quality eggs. What’s more, we got a similar number of upregulated and downregulated DEGs from low-quality vs. high-quality eggs. Function enrichment indicated that the RNA-level activities such as RNA binding and degradations were affected. This is inconsistent with our speculation that RNA-binding proteins play important roles in regulating RNA abundance and stability during the maturation of eggs. Further analysis of the upregulated and downregulated genes indicated that different levels of eggs exhibit the same function on RNAs by controlling different gene expressions during oogenesis. The DEGs from low-quality vs. high-quality eggs were enriched in the pathways of RNA degradation, oocyte meiosis, insulin signaling and p53 signaling, which were consistent with previous studies that these pathways were required for oocyte development [35–39].

Of all the DEGs identified from the low-quality vs. high-quality eggs, a small proportion was validated and reported to control egg quality as well as embryo development. Such as *igf2bp3* and *ythdf2*, have been revealed to maintain RNA stability and control the RNA degradation [22, 23, 40]. These two genes acted as potential markers of oocyte development and early embryonic development [21, 41]. In accordance with previous findings in Medaka [26], we found the expression level of *igf2bp3* was significantly increased in the low-quality eggs, indicating that the function of *igf2bp3* may be conserved in quality-control of eggs among different species. Thus, we considered *igf2bp3* as a potential marker of egg quality. Additionally, other important factors such as *sirt1* and *p53* are also essential factors for maintaining egg quality [42–44], they were both changed in expression between high-quality and low-quality eggs. Moreover, the transcriptomic comparison showed that other DEGs including RNA binding protein genes such as *elavl1*, *rbm25b* and *dnd1*, also involved in the pathways of oocyte and embryo development [45–49]. Notably, RNA binding protein gene *dnd1* was significantly increased in low-quality eggs, which has been reported as a germ plasm component to play an essential role in the survival of primordial germ cells (PGCs) [50, 51]. Meanwhile, the expression of some RBP genes, including *ddx4*, *piwil1* and *piwil2,* were associated with germ plasm assembly and were also perturbed in low-quality eggs. Combined with previous studies that the expression of germ plasm-associated genes may be related to female oocyte and embryo development, it’s implicit that the dysregulation of these genes leads to low-quality of egg [52, 53].

RNA stored in oocytes, including maternal RNAs, guides many fundamental processes of oogenesis and embryogenesis. However, the RNA turnover is controlled by RNA-binding proteins. Thus, RNA-binding proteins play an essential role in maintaining egg quality. In this study, through expression analysis, we identified thousands of regulators including RBPs and TFs. And further analyzed the contribution of these factors to egg quality. Results indicated that RBPs contributed more than the TFs (70.3% vs. 25.6%) in distinguishing the low and high-quality eggs. GO and KEGG results indicated that RBPs were significantly enriched at the pathways of RNA metabolism and oocyte development. This indicates that RBPs play a significant role in controlling egg quality via the regulation of RNA levels. Hence, the RBP expression level can reflect the egg quality in some extent. As mentioned above, Igf2bp3 is a representative member of RBP, has been well studied and acted as a regulator in controlling the RNA abundance, including maternal RNAs during oocyte development, and may be a novel biomarker of egg quality. But how Igf2bp3 and the other RBPs mediated RNA abundance and controlled egg quality remains to be elucidated. Overall, our results support that RNA-binding proteins control egg quality during oogenesis, but further studies on the mechanisms still need to be investigated.

## Conclusion

In this article, we revealed that maternal-inherited transcriptome could be used to indicate the developmental competence of eggs by our experimental strategy. While, We identified a group of differentially expressed genes associated with egg quality and found a cluster of RBP genes including *igf2bp3*, *zar1*, *elavl1*, *rbm25b* and related regulatory factors including *yy1*, *sirt1*, *anp32e*, *btg4* as novel biomarkers of egg quality.

## Methods

### Experimental fish and fertilization

All experiments procedures and animal handling were according to the requirements of the IACUC (the Institutional Animal Care and Use Committees) of Huazhong Agricultural University. XX female and XY male yellow catfish from the wild after three generation of selective breeding were bred and cultured on our own farm in Wuhan Hubei, China. We also obtained the permit of laboratory animals care and use from the laboratory animals center and college of fisheries of Huazhong Agricultural University. Sexually mature fish (one year) were selected for the experiment. All fish were maintained at 23–27 °C in a recirculating freshwater system. Artificial fertilization was performed during the mating season from June 2019 to July 2019. Testis separated from male yellow catfish after anesthesia were ground with sperm preservative solution (containing 63 mM NaCl, 19 mM KCl, 1.3 mM CaCl_2_, 4.7 mM MgSO_4_·7H_2_O, 2.5 mM NaHCO_3_, pH 7.4) to release milt, and the quality was determined by microscopic examination before insemination. Meanwhile, females were prepared by injecting 2 μg LHRH-A2 at 24 h pre-fertilization, and then injecting the mixture of 1 μg LHRH-A2 and 200 IU HCG at 12 h pre-fertilization. Eggs, obtained by gently squeezing the abdomen of mature females after anesthesia, were mixed with seminal plasma from the same male in 100 mm dishes (about 100 embryos/dish). After fertilization, embryonic development was tracked and the success rate was recorded. The fertilization rate, hatching rate and embryo malformation rate were recorded and calculated. The originally preserved eggs were classified into three levels, the low-quality, medium-quality, and high-quality groups (total 25 dishes divide into three groups).

### RNA-seq of eggs and data analysis

Total RNAs were extracted from unfertilized low-quality and high-quality eggs (about 25 eggs in each group) by TRIzol Reagent following the manufacturer’s instructions. The quality of total RNA was examined by 1% agarose gel electrophoresis. Only RNA samples of high quality were used for the cDNA synthesis. After purification, adapters were ligated to the cDNA and then amplified by PCR. cDNA libraries were built and the quality was assessed on the Agilent Bioanalyzer 2100. RNA-seq was performed on an Illumina NovaSeq 6000 platform with a 150 bp paired-end approach. Three biological replicates were sequenced for each group, respectively. For data analysis, fastp (v 0.23.0) [54] was firstly used to trim the low-quality bases and the adapters. Then clean reads were mapped to the yellow catfish reference genome using TopHat (v 2.0.13) [55]. Gene abundance estimation was performed using Cufflinks (v 2.2.1) [55] and indicated by FPKM (fragments per Kilobase of transcript per million reads mapped). Genes with mean FPKM > 1.0 in any group were retained for differential expression analysis using R package DESeq2 (v 1.34.0) [56]. Statistical comparisons were performed between low-quality and high-quality eggs. Thresholds of padj < 0.01 and |log2FoldChange| > 0.5 were used for filtering differentially expressed genes (DEGs). Gene Ontology (GO) analysis and Kyoto Encyclopedia of Genes and Genomes (KEGG) pathway enrichment were performed by R package clusterProfiler (v 4.2.0) [57, 58]. Heatmaps with k-means clustering were used to display gene expression patterns over groups of samples. The orthogonal partial least-squares discriminant analysis (OPLS-DA) model was built to screen species-specific genes that greatly contribute to the classification [59]. Gene expression levels from RNA-seq in this study were all according to the mean of FPKM in three replicates.

### Validation of gene expression by qRT-PCR

Total RNA was isolated from eggs of yellow catfish using TRIzol reagent (Invitrogen) and transcribed into cDNA using PrimeScript RT reagent kit with gDNA Eraser (Takara, RR047A). qRT-PCR was carried out using Hieff® qPCR SYBR® Green Master Mix (YEASEN) on a CFX Connect (Bio-rad) and mRNA expression was normalized to reference gene *actb1*. The data were analyzed using the 2^–ΔΔCt^ program. And the gene-specific primer sequences were listed in Table S1.

### Statistics

All statistical analyses of qRT-PCR and imaging were performed for technical replicates or experimental replicates. Student’s two-tailed unpaired t-test was used for statistical comparisons and data were shown as mean ± SD. *P* value of < 0.05 was considered as significant (*), while *P* < 0.01 and *P* < 0.001 as extremely significant (**) and (***), respectively.

## Supplementary Information

**Additional file 1: Fig. S1.** The Hatching rate and malformation rate in groups with different egg quality. **Fig. S2.** Function annotation of up and downregulated genes from low-quality eggs vs. high-quality eggs. **Fig. S3.** Function annotation of differential RBP genes from low-quality eggs vs. high-quality eggs. **Table S1.** The primers used for qRT-PCR.

## Data Availability

The datasets generated from the current study are available in the NCBI SRA repository, under project number PRJNA862635 (https://www.ncbi.nlm.nih.gov/sra/PRJNA862635).

## References

[1] Bobe J, Labbe C (2010). Egg and sperm quality in fish. Gen Comp Endocrinol.

[2] Brooks S, Tyler CR, Sumpter JP (1997). Egg quality in fish: what makes a good egg?. Rev Fish Biol Fish.

[3] Tagawa M, Hirano T (1987). Presence of thyroxine in eggs and changes in its content during early development of chum salmon, *Oncorhynchus keta*. Gen Comp Endocrinol.

[4] Izquierdo MS, Fernández-Palacios H, Tacon AGJ (2001). Effect of broodstock nutrition on reproductive performance of fish. Aquaculture.

[5] Pelegri F (2003). Maternal factors in zebrafish development. Dev Dyn.

[6] Aegerter S, Jalabert B, Bobe J (2005). Large scale real-time PCR analysis of mRNA abundance in rainbow trout eggs in relationship with egg quality and post-ovulatory ageing. Mol Reprod Dev.

[7] Bonnet E, Fostier A, Bobe J (2007). Microarray-based analysis of fish egg quality after natural or controlled ovulation. BMC Genomics.

[8] Mansour N, Lahnsteiner F, Patzner RA (2007). Distribution of lipid droplets is an indicator for egg quality in brown trout, *Salmo trutta* fario. Aquaculture.

[9] Ziv T, Gattegno T, Chapovetsky V, Wolf H, Barnea E, Lubzens E, Admon A (2008). Comparative proteomics of the developing fish (zebrafish and gilthead seabream) oocytes. Comp Biochem Physiol Part D Genomics Proteomics.

[10] Pan XF, Yang JX, Chen XY, Li ZY (2011). Broodstocks management, fecundity and the relationship between egg size and embryo survival ability of Sinocyclocheilus grahami. Dongwuxue Yanjiu.

[11] Devauchelle N, Alexandre JC, Le Corre N, Letty Y (1988). Spawning of turbot (Scophthalmus maximus) in captivity. Aquaculture.

[12] Forés R, Iglesias J, Olmedo M, Sánchez FJ, Peleteiro JB (1990). Induction of spawning in turbot (scophthalmus maximus L.) by a sudden change in the photoperiod. Aquac Eng.

[13] Lahnsteiner F, Patarnello P (2005). The shape of the lipid vesicle is a potential marker for egg quality determination in the gilthead seabream, Sparus aurata, and in the sharpsnout seabream, *Diplodus puntazzo*. Aquaculture.

[14] Ciereszko A, Wojtczak M, Dietrich GJ, Kuźmiński H, Dobosz S (2009). A lack of consistent relationship between distribution of lipid droplets and egg quality in hatchery-raised rainbow trout, *Oncorhynchus mykiss*. Aquaculture.

[15] Kopeika J, Kopeika E, Zhang T, Rawson DM, Holt WV (2003). Detrimental effects of cryopreservation of loach (Misgurnus fossilis) sperm on subsequent embryo development are reversed by incubating fertilised eggs in caffeine. Cryobiology.

[16] Avery TS, Killen SS, Hollinger TR (2009). The relationship of embryonic development, mortality, hatching success, and larval quality to normal or abnormal early embryonic cleavage in Atlantic cod, *Gadus morhua*. Aquaculture.

[17] Guzeloglu-Kayisli O, Kayisli UA, Taylor HS (2009). The role of growth factors and cytokines during implantation: endocrine and paracrine interactions. Semin Reprod Med.

[18] Adamczak R, Ukleja-Sokołowska N, Lis K, Dubiel M (2021). Function of follicular cytokines: roles played during maturation, development and implantation of embryo. Medicina (Kaunas).

[19] Sullivan CV, Chapman RW, Reading BJ, Anderson PE (2015). Transcriptomics of mRNA and egg quality in farmed fish: some recent developments and future directions. Gen Comp Endocrinol.

[20] Myers JN, Dyce PW, Chatakondi NG, Gorman SA, Quiniou SMA, Baofeng Su EP, Dunham RA, Butts IAE (2020). Analysis of specific mRNA gene expression profiles as markers of egg and embryo quality for hybrid catfish aquaculture. Comp Biochem Physiol A Mol Integr Physiol.

[21] Ivanova I, Much C, Di Giacomo M, Azzi C, Morgan M, Moreira PN, Monahan J, Carrieri C, Enright AJ, O'Carroll D (2017). The RNA m(6)a reader YTHDF2 is essential for the post-transcriptional regulation of the maternal transcriptome and oocyte competence. Mol Cell.

[22] Zhao BS, Wang X, Beadell AV, Lu Z, Shi H, Kuuspalu A, Ho RK, He C (2017). M(6)A-dependent maternal mRNA clearance facilitates zebrafish maternal-to-zygotic transition. Nature.

[23] Ren F, Lin Q (2020). Igf2bp3 maintains maternal RNA stability and ensures early embryo development in zebrafish. Commun Biol.

[24] Yang Y, Wang L, Han X, Yang WL, Zhang M, Ma HL, Sun BF, Li A, Xia J, Chen J (2019). RNA 5-Methylcytosine facilitates the maternal-to-zygotic transition by preventing maternal mRNA decay. Mol Cell.

[25] Takahashi K, Kotani T, Katsu Y, Yamashita M (2014). Possible involvement of insulin-like growth factor 2 mRNA-binding protein 3 in zebrafish oocyte maturation as a novel cyclin B1 mRNA-binding protein that represses the translation in immature oocytes. Biochem Biophys Res Commun.

[26] Wu X, Zhang Y, Xu S, Chang Y, Ye Y, Guo A, Kang Y, Guo H, Xu H, Chen L (2019). Loss of Gsdf leads to a dysregulation of Igf2bp3-mediated oocyte development in medaka. Gen Comp Endocrinol.

[27] Yu C, Ji SY, Sha QQ, Dang Y, Zhou JJ, Zhang YL, Liu Y, Wang ZW, Hu B, Sun QY (2016). BTG4 is a meiotic cell cycle-coupled maternal-zygotic-transition licensing factor in oocytes. Nat Struct Mol Biol.

[28] Yamamoto TM, Cook JM, Kotter CV, Khat T, Silva KD, Ferreyros M, Holt JW, Knight JD, Charlesworth A (2013). Zar1 represses translation in Xenopus oocytes and binds to the TCS in maternal mRNAs with different characteristics than Zar2. Biochim Biophys Acta.

[29] Miao L, Yuan Y, Cheng F, Fang J, Zhou F, Ma W, Jiang Y, Huang X, Wang Y, Shan L (2017). Translation repression by maternal RNA binding protein Zar1 is essential for early oogenesis in zebrafish. Development (Cambridge, England).

[30] Rong Y, Ji SY, Zhu YZ, Wu YW, Shen L, Fan HY (2019). ZAR1 and ZAR2 are required for oocyte meiotic maturation by regulating the maternal transcriptome and mRNA translational activation. Nucleic Acids Res.

[31] Pan Z, Zhu C, Chang G, Wu N, Ding H, Wang H (2021). Differential expression analysis and identification of sex-related genes by gonad transcriptome sequencing in estradiol-treated and non-treated Ussuri catfish Pseudobagrus ussuriensis. Fish Physiol Biochem.

[32] Ljubobratović U, Kwiatkowski M, Tóth F, Demény F (2021). Effects of hormonal treatment before water warming on synchronisation of spawning time, oocyte size, and egg quality in pikeperch (Sander lucioperca). Anim Reprod Sci.

[33] Kottmann JS, Tveiten H, Miest JJ, Tomkiewicz J (2021). Sex steroid dynamics and mRNA transcript profiles of growth- and development-related genes during embryogenesis following induced follicular maturation in European eel. Gen Comp Endocrinol.

[34] Harel M, Tandler A, Kissil GW, Applebaum SW (1994). The kinetics of nutrient incorporation into body tissues of gilthead seabream (Sparus aurata) females and the subsequent effects on egg composition and egg quality. Br J Nutr.

[35] Cabrera-Quio LE, Schleiffer A, Mechtler K (2021). Zebrafish Ski7 tunes RNA levels during the oocyte-to-embryo transition. PLoS Genet.

[36] Jin X, Wang K, Wang L, Liu W, Zhang C, Qiu Y, Liu W, Zhang H, Zhang D, Yang Z (2022). RAB7 activity is required for the regulation of mitophagy in oocyte meiosis and oocyte quality control during ovarian aging. Autophagy.

[37] Templeman NM, Luo S, Kaletsky R, Shi C, Ashraf J, Keyes W, Murphy CT (2018). Insulin signaling regulates oocyte quality maintenance with age via Cathepsin B activity. Curr Biol.

[38] Chen HL, Cheng JY, Yang YF, Li Y, Jiang XH, Yang L, Wu L, Shi M, Liu B, Duan J (2020). Phospholipase C inhibits apoptosis of porcine oocytes cultured in vitro. J Cell Biochem.

[39] Tang L, Chen J, Ye Z, Zhao M, Meng Z, Lin H, Li S (2019). Transcriptomic analysis revealed the regulatory mechanisms of oocyte maturation and hydration in Orange-spotted grouper (Epinephelus coioides). Marine Biotechnol (New York, NY).

[40] Lin Tang JC, Ye Z, Zhao M, Meng Z, Lin H, Li S, Zhang Y (2019). Transcriptomic analysis revealed the regulatory mechanisms of oocyte maturation and hydration in Orange-spotted grouper (Epinephelus coioides). Mar Biotechnol (New York, NY).

[41] Räty M, Ketoja E, Pitkänen T, Ahola V, Kananen K, Peippo J (2011). In vitro maturation supplements affect developmental competence of bovine cumulus-oocyte complexes and embryo quality after vitrification. Cryobiology.

[42] Iljas JD, Wei Z (2020). Sirt1 sustains female fertility by slowing age-related decline in oocyte quality required for post-fertilization embryo development. Aging Cell.

[43] Levine AJ, Tomasini R, McKeon FD, Mak TW, Melino G (2011). The p53 family: guardians of maternal reproduction. Nat Rev Mol Cell Biol.

[44] Chakravarti A, Thirimanne HN, Brown S, Calvi BR (2022). Drosophila p53 isoforms have overlapping and distinct functions in germline genome integrity and oocyte quality control. Elife.

[45] Calder MD, Madan P, Watson AJ (2008). Bovine oocytes and early embryos express Staufen and ELAVL RNA-binding proteins. Zygote (Cambridge, England).

[46] Colegrove-Otero LJ, Devaux A, Standart N (2005). The Xenopus ELAV protein ElrB represses Vg1 mRNA translation during oogenesis. Mol Cell Biol.

[47] Peshkin L, Wühr M, Pearl E, Haas W, Freeman Robert M, Gerhart John C, Klein Allon M, Horb M, Gygi Steven P, Kirschner Marc W (2015). On the relationship of protein and mRNA dynamics in vertebrate embryonic development. Dev Cell.

[48] Bhattacharya C, Aggarwal S, Zhu R, Kumar M, Zhao M, Meistrich ML, Matin A (2007). The mouse dead-end gene isoform alpha is necessary for germ cell and embryonic viability. Biochem Biophys Res Commun.

[49] Li Q, Li Y, Yang S, Huang S, Yan M, Ding Y, Tang W, Lou X, Yin Q, Sun Z (2018). CRISPR-Cas9-mediated base-editing screening in mice identifies DND1 amino acids that are critical for primordial germ cell development. Nat Cell Biol.

[50] Yamaji M, Jishage M, Meyer C, Suryawanshi H, Der E, Yamaji M, Garzia A, Morozov P, Manickavel S, McFarland HL (2017). DND1 maintains germline stem cells via recruitment of the CCR4-NOT complex to target mRNAs. Nature.

[51] Weidinger G, Stebler J, Slanchev K, Dumstrei K, Wise C, Lovell-Badge R, Thisse C, Thisse B, Raz E (2003). Dead end, a novel vertebrate germ plasm component, is required for zebrafish primordial germ cell migration and survival. Curr Biol.

[52] Yang CX, Wright EC, Ross JW (2012). Expression of RNA-binding proteins DND1 and FXR1 in the porcine ovary, and during oocyte maturation and early embryo development. Mol Reprod Dev.

[53] Aguero T, Kassmer S, Alberio R, Johnson A, King ML (2017). Mechanisms of vertebrate germ cell determination. Adv Exp Med Biol.

[54] Chen S, Zhou Y, Chen Y, Gu J (2018). Fastp: an ultra-fast all-in-one FASTQ preprocessor. Bioinformatics (Oxford, England).

[55] Trapnell C, Roberts A, Goff L, Pertea G, Kim D, Kelley DR, Pimentel H, Salzberg SL, Rinn JL, Pachter L (2012). Differential gene and transcript expression analysis of RNA-seq experiments with TopHat and cufflinks. Nat Protoc.

[56] Love MI, Huber W, Anders S (2014). Moderated estimation of fold change and dispersion for RNA-seq data with DESeq2. Genome Biol.

[57] Yu GC, Wang LG, Han YY, He QY (2012). clusterProfiler: an R package for comparing biological themes among gene clusters. Omics.

[58] Ogata H, Goto S, Sato K, Fujibuchi W, Bono H, Kanehisa M (1999). KEGG: Kyoto encyclopedia of genes and genomes. Nucleic Acids Res.

[59] Kang C, Zhang Y, Zhang M, Qi J, Zhao W, Gu J, Guo W, Li Y (2022). Screening of specific quantitative peptides of beef by LC-MS/MS coupled with OPLS-DA. Food Chem.

