# Supplementary material for: RNA binding proteins are potential novel biomarkers of egg quality in yellow catfish
Source: BMC Genomics. 2023 Mar 16;24:121. doi: 10.1186/s12864-023-09220-9 (PMC10018890; doi:10.1186/s12864-023-09220-9)
Supplement: Supplementary file 1 — Additional file 1: Fig. S1. The Hatching rate and malformation rate in groups with different egg quality. Fig. S2. Function annotation of up and downregulated genes from low-quality eggs vs. high-quality eggs. Fig. S3. Function annotation of differential RBP genes from low-quality eggs vs. high-quality eggs. Table S1. The primers used for qRT-PCR. [file 12864_2023_9220_MOESM1_ESM.pdf]

Supplemental figure 1

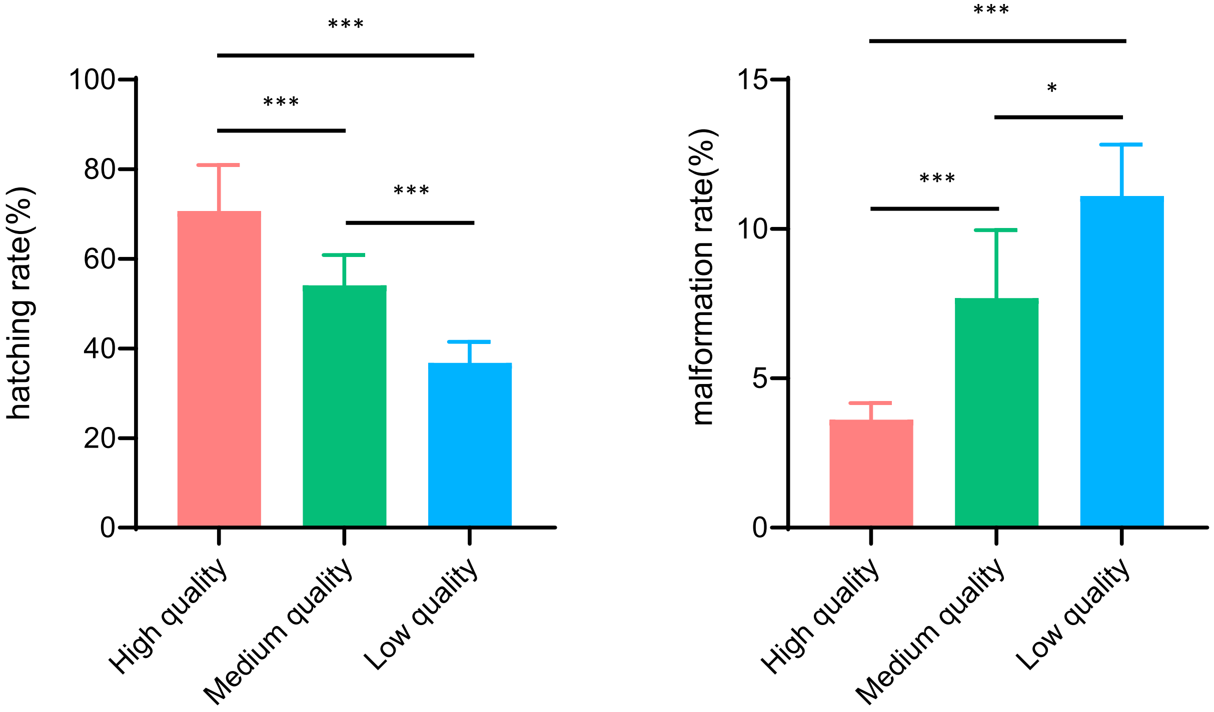

Supplemental figure 2

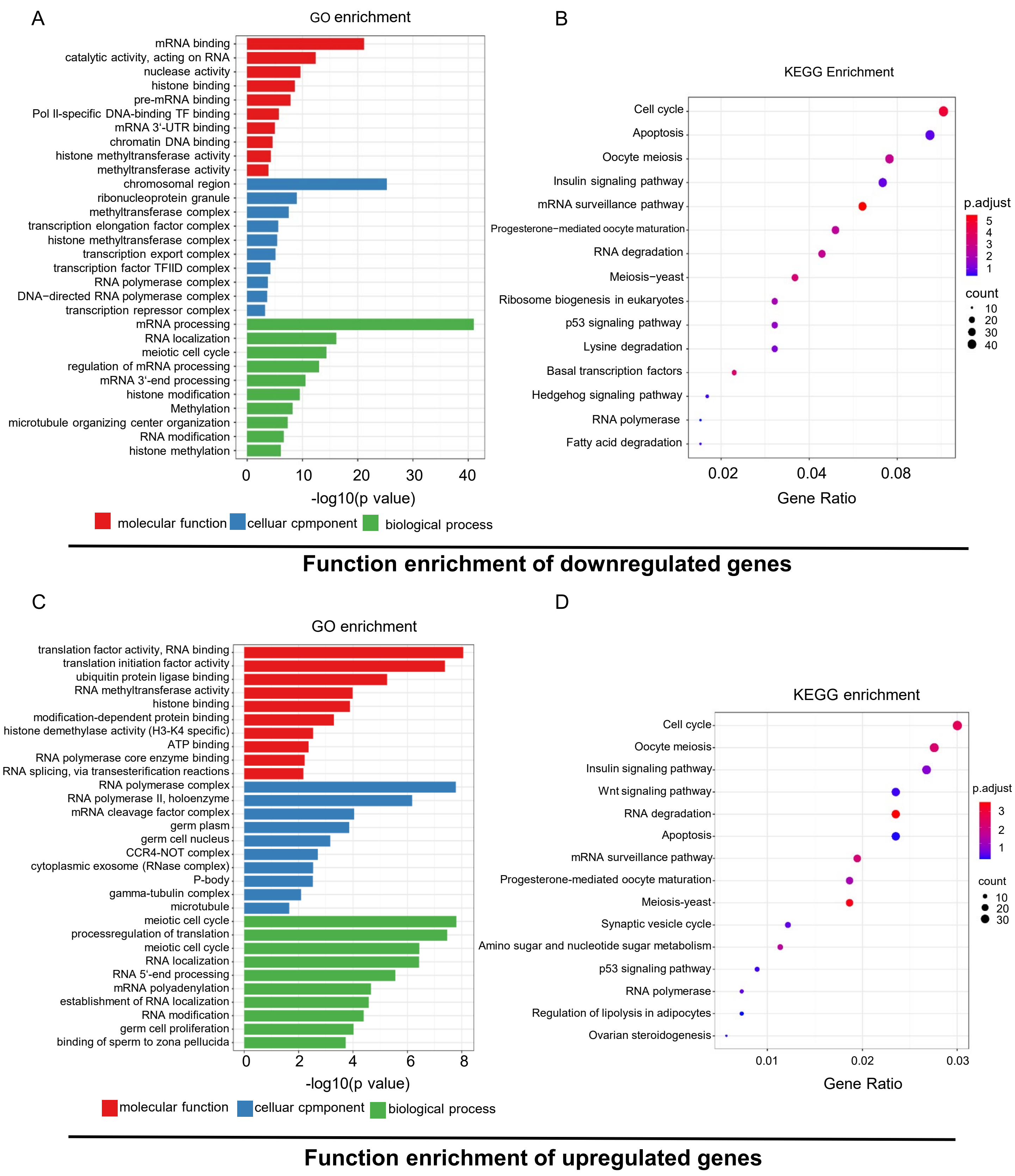

Supplemental figure 3

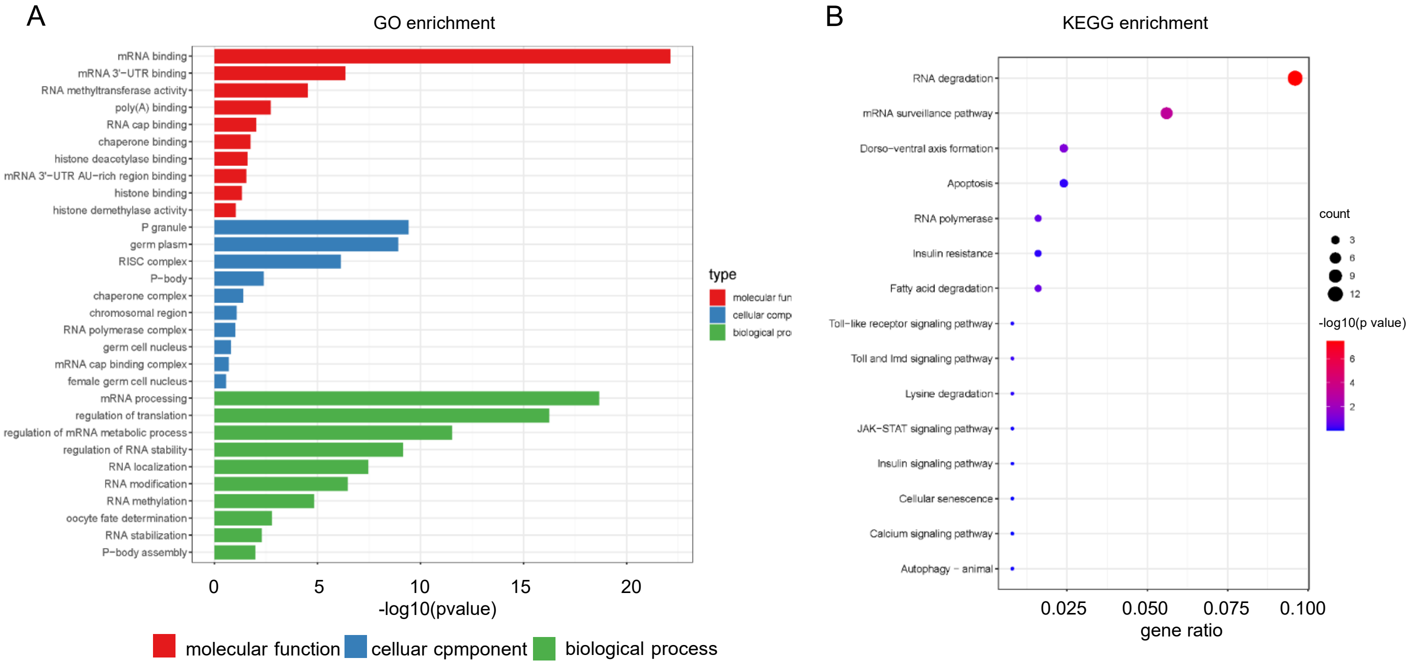

Function enrichment of upregulated genes

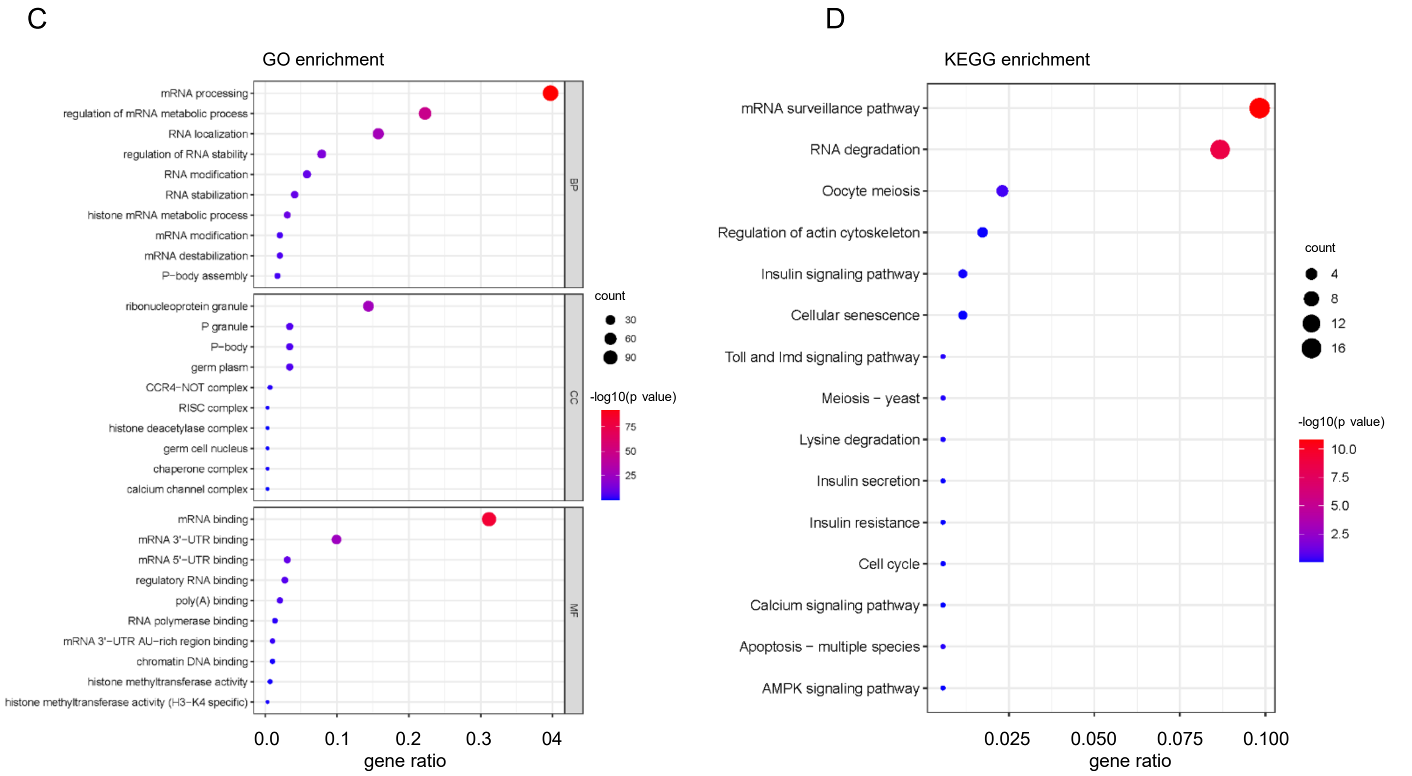

Function enrichment of downregulated genes

Supplemental figure legends

Figure S1. The Hatching rate and malformation rate in groups with different egg quality.

Hatching rate and malformation rate were analysed.

Figure S2. Function annotation of up and downregulated genes from low-quality eggs vs. high-quality eggs.

(A) GO enrichment of downregulated genes.

(B) KEGG enrichment of downregulated genes.

(C) GO enrichment of upregulated genes.

(D) KEGG enrichment of upregulated genes.

Figure S3. Function annotation of differential RBP genes from low-quality eggs vs. high-quality eggs.

(A) GO enrichment of upregulated RBP genes.

(B) KEGG enrichment of upregulated RBP genes.

(C) GO enrichment of downregulated RBP genes.

(D) KEGG enrichment of downregulated RBP genes.

Supplemental table

anp32e-f	ACAATAACATCTCAGGCTCT
anp32e-r	ACAATTAAAAAGGTCCAGGC
rbm25b-f	GCAGCCCTAGTCAACCAAGT
rbm25b-r	CCACGACAGACCAGTCAAGC
kiaa0101-f	TGGAGACTTCTTCGGTGGC
kiaa0101-r	GGACTTCCTGGGTGCTTTG
elavl1a-f	GTGAGCAGGAAACTCCGTCT
elavl1a-r	TGATTAGGATTCGCAGCAAA
igf2bp3-f	ATGAGACTGTGCTGTTTGAT
igf2bp3-r	GTTTGGTGATATTGCGGATG
zar1-f	AGTGCGTCAGGCGGCTAAG
zar1-r	AATAACCCTCACCGTCTCCAT
sirt1-f	CAGTCCAATCAGTAATCGCC
sirt1-r	TCTGTCTCTCCAACACCACC
kdm2a-f	ACAGAACGAAGAGCGGGAAAC
kdm2a-r	CGGTGGGCGTAGCAAGGAC
β-actin-f	TCCCATCCATTGTTGGTCGC
β-actin-r	ATCTTTTCCCTGTTGGCTTT
yy1-f	GATGCGACGACTCGGACCTT
yy1-r	TGCCGCTTCCTTTCTTCA
p53-f	TGGGAAAACGAAGAGCAA
p53-r	AATCGGAGGTGACAGGGAC
btg4-f	GTTGGAGGGGAAAAGGGGAGA
btg4-r	TTGGGATTGGAGACCGTGGG
kdm2b-f	GCCTGGTTGGCTATTTTC
kdm2b-r	ACTCAGCATCAGAGGTGT
dnd1-f	GAACCGTGGCTTTGCTT
dnd1-r	TGCCTCTTCTCCGTGCT
ythdf2-f	ACGACATCCATCGCTCAA
ythdf2-r	AACCGACCCTTCCACTTAT

Table S1. **The primers used for qRT-PCR.**

Primers of qRT-PCR used for validation of differently expressed genes from low-quality eggs vs. high-quality eggs.
